# Remarkable improvement in a very severe chronic obstructive pulmonary disorder patient after use of noninvasive intermittent positive pressure ventilator: A case report

**DOI:** 10.1097/MD.0000000000012877

**Published:** 2018-10-26

**Authors:** Ji Ho Jung, Young Ho Lim, Jang Woo Lee, Won Ah Choi, Mi Ri Suh, Seong-Woong Kang

**Affiliations:** a Department of Rehabilitation Medicine and Rehabilitation Institute of Neuromuscular Disease, Gangnam Severance Hospital, Yonsei University College of Medicine, Seoul; b Pulmonary Rehabilitation Center, Gangnam Severance Hospital, Seoul; c Department of Physical Medicine and Rehabilitation, Hallym University Sacred Heart Hospital, Hallym University College of Medicine, Anyang; d Department of Physical Medicine and Rehabilitation, National Health Insurance Service Ilsan Hospital, Goyang, Korea.

**Keywords:** activities of daily living, chronic obstructive pulmonary disease, intermittent positive-pressure ventilation, noninvasive ventilation, respiratory function tests

## Abstract

**Rational:**

Chronic obstructive pulmonary disease (COPD) impairs lung function and induces systemic effects, resulting in impaired quality of life. Skeletal muscle dysfunction—characteristic of advanced COPD patients—limits a patient's ability to perform activities of daily living (ADL). In addition, dysphagia is commonly observed in COPD patients.

**Patient concern:**

This case report documents a 42-year-old man with very severe COPD. He experienced aggravation of the symptoms during standard medical treatment and his ability to perform the ADL was significantly impaired. Furthermore, his dysphagia worsened despite oromotor training.

**Diagnosis:**

He was diagnosed as very severe COPD have a problem with swallowing and respiratory function.

**Intervention:**

Upon NIPPV treatment, the patient's ability to perform the ADL, as well as his dysphagia, showed improvement.

**Outcomes:**

Thus, we report the remarkable improvement of physical function, as well as dysphagia, in a very severe COPD patient after NIPPV treatment.

**Lessons:**

NIPPV may be useful as a treatment option for such patients.

## 1 Introduction

Chronic obstructive pulmonary disease (COPD) is known to induce—in addition to respiratory symptoms—a decrease in muscle strength and endurance caused by systematic effects, as well as vulnerability to fatigue, and a decline in exercise capacity and swallowing function.^[1,2]^ A decrease in physical activities, and the resulting sedentary lifestyle, further exacerbate the weakened pulmonary function, resulting in a vicious cycle that leads to the decline of health-related quality of life (HRQOL) and physical ability at a rate that is disproportionate to the decline of the lung function. COPD is not simply a lung disorder; COPD-related mortality is associated with deterioration in a patient's overall physical function. ^[3]^

In acute exacerbation of COPD, noninvasive positive pressure ventilation (NIPPV) is known to improve related symptoms and to reduce the length of hospital stay, as well as intubation rate and mortality rate.^[4]^ If NIPPV is utilized as adjunct treatment for severe COPD patients, modest improvement in exercise performance may be expected; however, the level of evidence supporting this is low and it remains categorized as a weak recommendation.^[5]^

This case report seeks to document a dramatic improvement in various physical functions, as observed in a GOLD criteria stage IV (very severe) COPD patient after nocturnal NIPPV treatment. Moreover, this is the first report on the improvement of swallowing function upon nocturnal NIPPV treatment. Patient had provided informed consent for the case report and agreed publishing.

## 2 Case report

The patient was diagnosed with acute myeloid leukemia in 1994 at the age of 19 and underwent bone marrow transplantation. As a sequela of the graft versus host disease after the transplantation, he exhibited symptoms of dyspnea in 1995 and was diagnosed with COPD in 1996. The patient regularly underwent medical treatment, but the dyspnea continued to exacerbate. As the resting state oxygen saturation level decreased to <90%, long-term oxygen therapy was commenced in September 2015. Dyspnea and deterioration of physical activity continued to worsen. Beginning in June 2016, he was nearly sedentary and suffered from swallowing difficulty, accompanied by frequent symptoms of aspiration. In November 2016, he was referred to our department for the evaluation of dysphagia. At that time, although his manual muscle power was good in both upper and lower extremities without a focal neurologic deficit, he could not walk more than 3 to 4 m independently. On arterial blood gas analysis, *p*CO_2_ was approximately 70 mm Hg. A videofluoroscopic swallowing study (VFSS) performed on November 18 revealed delayed oral transit time, impaired laryngeal elevation and swallowing reflex, inadequate upper esophageal sphincter (UES) opening, and aspiration of liquid. Rehabilitative treatment of dysphagia was commenced; however, the follow-up VFSS, performed on November 29 and December 20 of 2016, showed continued aggravation, despite a medically stable condition and regular oromotor training. No abnormal findings were observed in the brain magnetic resonance imaging or electrodiagnostic study, which were conducted to identify any other causes of dysphagia.

The patient was admitted to our department on December 28, 2016, for consideration of NIPPV. The transcutaneous *p*CO_2_ monitoring, performed overnight via V-Sign Sensor (SenTec Digital Blood Gas Monitoring System; Sentec AG, Therwil, Switzerland), indicated a maximal *p*CO_2_ of 72.8 mm Hg and a mean *p*CO_2_ of 62.4 mm Hg. Nocturnal NIPPV, with the mode of bilevel positive airway pressure, was then applied via a nasal mask; *p*CO_2_ monitoring conducted with the ventilator on January 6, 2017, showed an improvement, indicating a maximal *p*CO_2_ of 50.8 mm Hg and a mean *p*CO_2_ of 47 mm Hg. After undergoing NIPPV treatment, the patient showed a remarkable improvement in physical function. The patient could not even perform a 6-minute walking test (6MWT) in August and November 2016; however, on February 28, 2017, he completed a 490-m walk and SpO_2_ was measured at 94% after the examination, without oxygen supply. In examinations, such as the Brief Fatigue Inventory (BFI), COPD Assessment Test, and Modified Fatigue Impact Scale (MFIS), the patient also showed improvements in COPD symptoms and fatigue-related indicators. These improvements led to enhancement in activities of daily living (ADL), resulting a remarkable improvement in indices such as the Functional Independence measure (FIM) and London Chest Activity of Daily Living (LCADL) test (Table 1).

**Table 1:**
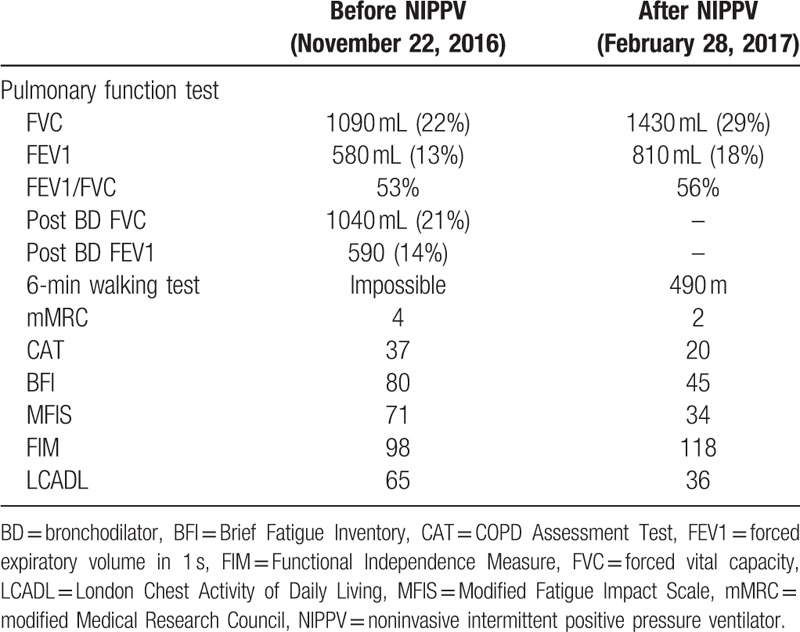
Comparison of various parameters before and after application of NIPPV.

The patient was readmitted to our department on March 15, 2017. During transcutaneous *p*CO_2_ monitoring with nocturnal NIPPV, the patient showed improvement, with a maximal *p*CO_2_ of 37.7 mm Hg and a mean *p*CO_2_ of 35.8 mm Hg. The follow-up VFSS showed an improvement, compared with the previous study; only penetration was observed when swallowing liquids. During this period, medications related to COPD were not changed.

## 3 Discussion

The effect of applying NIPPV in COPD patients has been proven. It corrects acute respiratory acidosis and relieves dyspnea during acute exacerbation, leading to a lowered rate of ventilator-associated pneumonia and extended hospital stays that often result in mortality. Long-term application of NIPPV may also improve survival rate and quality of life.^[4]^ However, in regards to physical function, inconsistent results have been reported, especially in severe stages. In previous studies, an improvement of exercise tolerance after the application of NIPPV was only found in mild or moderate COPD. Unlike previous studies, we found an improvement in exercise tolerance, as measured by the 6MWT, in a severe COPD patient.^[5,6]^ We found both improvement of physical function and of exercise tolerance after applying NIPPV; there are many factors that can affect this dramatic improvement, including the patient's relatively young age. Importantly, there has been no previous study showing dramatic improvement of physical function in a severe COPD patient solely after NIPPV.

Fatigue is one of the typical symptoms among COPD patients, which exacerbates as COPD progresses. Abundant evidence shows that dysfunction of the skeletal muscles in COPD patients contributes to such fatigue. In the case of lower extremity muscles, the proportion of type II fibers becomes increased, resulting in vulnerability to fatigue in the lower extremities. These findings are more pronounced in patients with severe COPD.^[7]^ The patient in this study also had experienced a very severe COPD state for a long period, during which the lower extremity muscles had become particularly vulnerable, such that the distance of walking became gradually shortened. Studies have shown that the application of NIPPV to patients with severe COPD elicits improvement in various indices; however, there is no clear effect on exercise endurance.^[5,6]^ In this case, NIPPV treatment improved muscle fatigue, which was meaningful in that it led to dramatic improvement of the ability to perform the ADL. The BFI and MFIS (fatigue-related questionnaires) indicated dramatic recovery after NIPPV treatment, which induced improvements in both ambulatory function and in the ADL. This was further manifested by changes in the FIM and LCADL indices.

A previous study has shown that the overall survival rate and HRQOL were significantly improved among GOLD stage IV COPD patients to whom long-term NIPPV was applied in addition to the standard treatment; however, there was no significant difference in the results of the 6MWT.^[6]^ The patient in this study, unlike in the previous studies, clearly showed remarkable improvement in his exercise capacity. There are 2 major differences between the patient in this case and the patient group in the prior study. First, the subjects recruited in the prior study were clinically stable. In this case report, however, the patient underwent drastic exacerbation of dyspnea and pulmonary functions during the 6 months preceding NIPPV treatment. Further, there is an age difference of approximately 20 years between the group in the prior study (mean age, 62.2 years) and this case patient (42 years). Considering these differences, NIPPV treatment may be expected to improve the exercise capacities of a relatively young patient with a marked deterioration of symptoms and function, compared with a stable old patient.

According to a recent review article, various mechanisms have been suggested with respect to the causes of increased dysphagia in COPD patients.^[8]^ However, no study has been published showing that the use of NIPPV is related to remarkable improvements in swallowing function; to our knowledge, this case report is the first to document improvement in dysphagia after nocturnal NIPPV.

## 4 Conclusion

We observed improvement in the exercise capacity, restored independence in the ADL, and recovery of dysphagia, when respiratory support with NIPPV alone was applied to a very severe COPD patient with hypercapnia. Therefore, NIPPV may be useful as a treatment option for such patients.

## Author contributions

**Conceptualization:** Won Ah Choi, Mi Ri Suh.

**Data curation:** Jangwoo Lee.

**Methodology:** Jangwoo Lee.

**Supervision:** Seong-Woong Kang, Eunyoung Kim.

**Writing – original draft:** JIHO JUNG.

**Writing – review & editing:** Young Ho Lim.

Seong-Woong Kang orcid: 0000-0002-7279-3893.
